# Fuzzy Computing Model of Activity Recognition on WSN Movement Data for Ubiquitous Healthcare Measurement

**DOI:** 10.3390/s16122053

**Published:** 2016-12-03

**Authors:** Shu-Yin Chiang, Yao-Chiang Kan, Yun-Shan Chen, Ying-Ching Tu, Hsueh-Chun Lin

**Affiliations:** 1 Department of Information and Telecommunications Engineering, Ming Chuan University, Gui-Shan, Taoyuan 333, Taiwan; sychiang@mail.mcu.edu.tw (S.-Y.C.); conanto1986@hotmail.com (Y.-C.T.); 2 Department of Communications Engineering, Yuan Ze University, Chung-Li, Taoyuan 320, Taiwan; yckan@saturn.yzu.edu.tw; 3 Department of Physical Therapy, China Medical University, 91 Hsueh-Shi Road, Taichung 40402, Taiwan; u103009405@cmu.edu.tw; 4 Department of Health Risk Management, China Medical University, 91 Hsueh-Shi Road, Taichung 40402, Taiwan

**Keywords:** WSN, accelerometer, gyroscope, activity recognition, neuro fuzzy, ubiquitous health care

## Abstract

Ubiquitous health care (UHC) is beneficial for patients to ensure they complete therapeutic exercises by self-management at home. We designed a fuzzy computing model that enables recognizing assigned movements in UHC with privacy. The movements are measured by the self-developed body motion sensor, which combines both accelerometer and gyroscope chips to make an inertial sensing node compliant with a wireless sensor network (WSN). The fuzzy logic process was studied to calculate the sensor signals that would entail necessary features of static postures and dynamic motions. Combinations of the features were studied and the proper feature sets were chosen with compatible fuzzy rules. Then, a fuzzy inference system (FIS) can be generated to recognize the assigned movements based on the rules. We thus implemented both fuzzy and adaptive neuro-fuzzy inference systems in the model to distinguish static and dynamic movements. The proposed model can effectively reach the recognition scope of the assigned activity. Furthermore, two exercises of upper-limb flexion in physical therapy were applied for the model in which the recognition rate can stand for the passing rate of the assigned motions. Finally, a web-based interface was developed to help remotely measure movement in physical therapy for UHC.

## 1. Introduction

Modern health care services are experiencing new challenges in the rapid growth of long term illnesses and ethical demands in health care. Ubiquitous health monitoring technique offers self-management solutions for older adults and disabled patients who need regular healthcare or rehabilitation at home. Activity detection is an important issue in ubiquitous health care (UHC), particularly for daily monitoring of patients with motor handicaps. In pursuit of rehabilitation for these patients, specific motions and facilities are prescribed in routine and continuous programs during the recovery period [1,2,3]. If physiatrists can completely review the daily records of patients through UHC with ambulatory measurements, they can design more helpful therapies [4,5]. Therefore, activity recognition is the prospective solution to accomplish this task.

Appropriate techniques have been used to capture and recognize movements of people, such as lying down, sitting, standing, and walking [6]. Typically, image [7] and non-image [8] procedures have been extensively used to classify movements of the human body. Thus, patients would prefer non-image UHC devices because of privacy, convenience, and cost of equipment [9]. Requirements of UHC in relation to rehabilitation are expanded by the concept of homecare personal area networks that provide ambulatory monitoring and supporting hospital functions by using ad hoc or body area networks [10,11,12,13,14,15,16,17,18]. In the past decade, wireless sensor networks (WSNs) have been gradually deployed and applied to detect various physiological signals because of micro-electro-mechanical system (MEMS), integrated circuits (IC), and rapid advances in radio frequency (RF) [19,20]. These types of technologies have been extended to different UHC devices to measure the blood pressure of patients and monitor variations of their movements and environment at home [21,22]. Numerous studies promoted these emerging techniques in various home health care systems because of their long durability, low power, and mobile capacity for patients to ubiquitously communicate health care information and clinical data to the hospital [23,24,25,26]. For this approach to physical therapy, several activity recognition procedures were examined to improve motor measurements with accelerometers or gyroscopes for further WSN-enabled UHC applications to prevent unnecessary videotaping or photographing [27,28].

Computing algorithms such as fuzzy logic, artificial neural network (ANN), and backward propagation neural network (BPNN) were popular for recognizing and classifying features of motions in past studies. For example, rule-based fuzzy logic enables classification of regular gesture patterns [29,30,31]; the ANN with neural-fuzzy functions retains training capability to recognize irregular movements [32]; the BPNN follows the ANN with three layers of machine learning [33]. Thus, fuzzy inference system (FIS) based on Zadeh’s fuzzification algorithms that map the functioned features from an input space to an output space can comprise a list of if-then rules for complex controls and decision processes [34]. In which, two well established types of FIS can be utilized: i.e., the Mamdani type which uses typical membership functions of output features for defuzzification [35] and the Sugeno type which entails weight average by constant or linear expression to compute crisp output [36]. The FIS was employed in many studies to distinguish patterns of human activities that were measured by various mobile devices with an approximate 90%–100% recognition rate dependent on features [37,38,39,40,41]. In addition, the neuro-fuzzy algorithm was suggested to adapt the training-based dataset to the training procedure for comprehensive and accurate recognition of adopted features [42,43,44,45].

This study established the activity recognition model for the self-designed wearable WSN sensor, which combines MEMS-based accelerometer and gyroscope chips to carry six motion components of acceleration and angular velocity for detecting body movement [46]. The subjects wore two WSN sensors, one on the chest and the other one on the thigh, to transmit the required signals by using a convenient measurement procedure. The WSN motes deployed in a space of a body area network could transport sensed data to the lab while users wore the WSN sensors in the other room. The measurement data were sent to the backend server of the monitoring system through a WSN gateway that was created in our previous work [47]. The motion components can be the potential feature sets in the FIS to calculate the combinative data of activity recognition. In this paper, the WSN sensor configuration is briefly described in the next section, and is followed by a presentation of the measurement methods. The FIS designs with fuzzification and defuzzification are then processed to compute the sensed data input, and to identify the output movements. The procedure is further practiced by two upper-limb flexion actions to determine a threshold of the specific activity in physical therapy for UHC measurement. Finally, the results are discussed, and concluding remarks are presented.

## 2. Measurement Methods

One of UHC’s programs in physical therapy is enabling physiatrists to remotely monitor movements of the patients when they need to continuously rehabilitate their disabilities at home. The prior studies measured acceleration data to discover the activity recognition algorithms [48,49]. Thus, the gyroscope and accelerometer can return signals related to angular velocity and acceleration, respectively, and detect the motions like *lie*, *sit*, *stand*, *walk*, and *run*. The measured data can be converted as the components in spatial coordinates to denote the specific movements. Both of the MEMS-enabled chips are extensively embedded within a portable device to sense positions of the subjects when they are worn by the users. As transporting data through the WSN to the backend system, the fuzzy inference procedure for activity recognition that is supported by the Fuzzy Logic toolbox of MATLAB™ (by The MathWorks Inc., Natick, MA, USA) can enable the UHC application in physical therapy.

### 2.1. WSN Sensors and Signals

**Figure 1:**
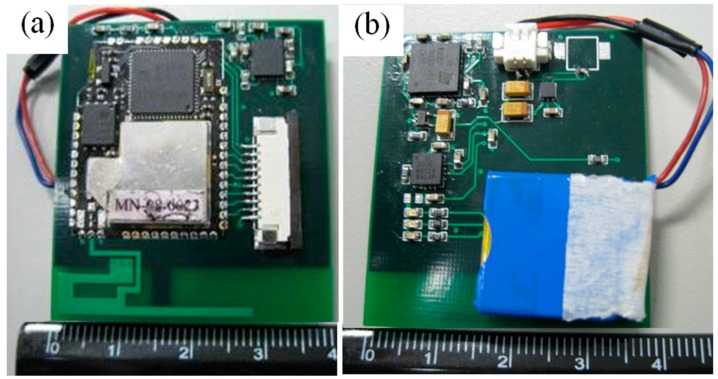
The wearable WSN body motion sensor with inertial sensing node: (**a**) WSN mote; (**b**) accelerometer and gyroscope modules with rechargeable battery.

The WSN sensor was integrated within a mote that configures the embedded micro control unit (MCU), RF, and antenna, as shown in Figure 1a, to deliver signals of the wireless sensor. The accelerometer and gyroscope were installed in an inertial sensing node as shown in Figure 1b to detect six components of acceleration and angular velocity. The sensor modules are designed with low-power emissions for safety, and are compliant with TinyOS which is the open-source embedded operating system available for remote control through the typical network protocol. In the design, a packet filtering module was created at the receiver sink of the backend server to convert measured signals into physical values.

According to the design specifications, the self-developed MEMS-based wearable device allows measuring acceleration and angular velocity respectively within ±10 g and ±1000°/s. Besides, the problem of noise for low gravity was experienced and calibrated in the development of our previous work [41] to ensure the fidelity of the device in measurement. With this concern, the relative values of measured data with respect to initial data, instead of the absolute values, are suggested for calibration.

**Figure 2:**
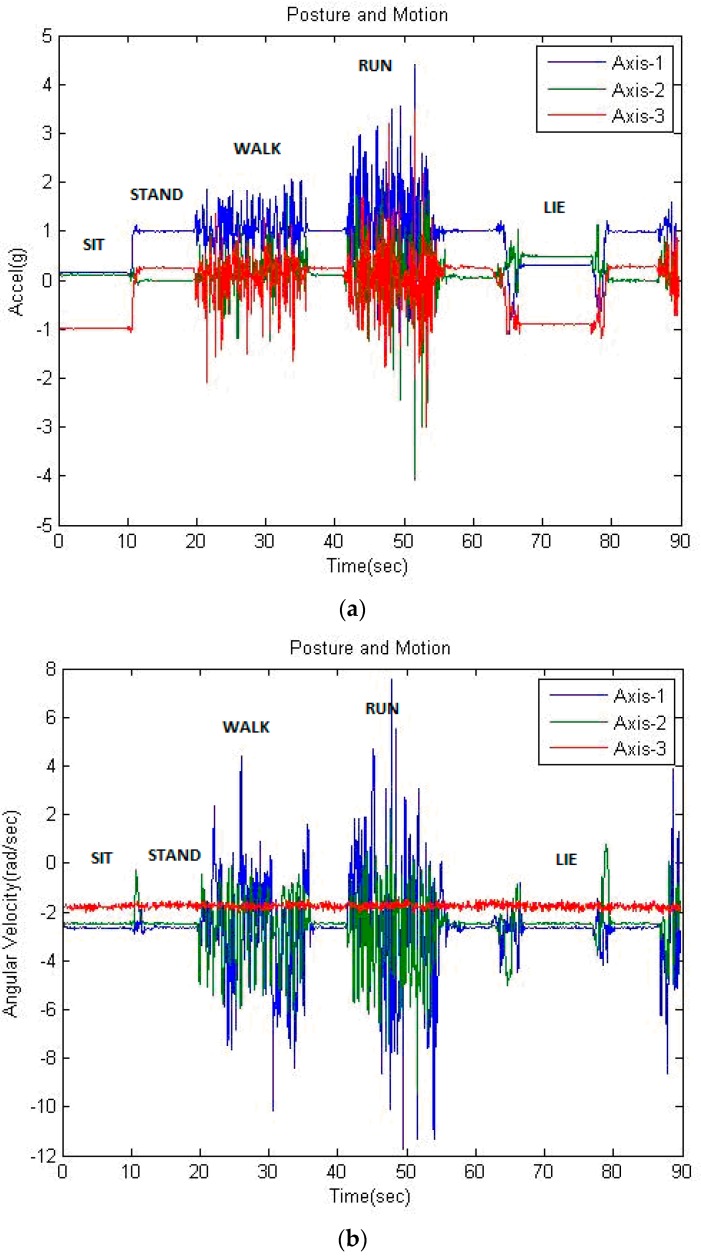
Variations of acceleration in gravity (**a**) and angular velocity in degree/sec (**b**) detected by mote A.

The five basic motions above cover the body movement including regular sway of the upper and lower limbs. In which, the potential motor features can be observed in movements of the chest and thigh portions for motions of the upper and lower body. We thus used two WSN sensors to measure the specific motor features of basic motions. The subject wore sensors A and B on the right chest and the left thigh, respectively, to acquire the components of acceleration and angular velocity. The variations of retrieved data can be plotted in the diagrams, as shown in Figure 2, for recognition. According to manufacture specifications, we located the local coordinate system for three sets of data packets of either accelerometer or gyroscope along 1-, 2-, and 3-axes of the device. In which, the 3-axis is oriented for gravity. We further defined the global coordinate system of *x*-, *y*-, and *z*-axes as vertical to the ground, lateral to the body, and forward/backward, respectively. The static posture “stand” is set as the initial status of measurement (i.e., the 1-axis of both sensors is vertical to the ground in the beginning). Let *g_A_*, *g_B_*, *ω_A_*, and *ω_B_* be the vectors of acceleration and angular velocity measured by sensors A and B, respectively. Each vector consists of three components in the local coordinate; that is, *g_A_* = (*g_A_*_1_, *g_A_*_2_, *g_A_*_3_), *g_B_* = (*g_B_*_1_, *g_B_*_2_, *g_B_*_3_), *ω_A_* = (*ω_A_*_1_, *ω_A_*_2_, *ω_A_*_3_), and *ω_B_* = (*ω_B_*_1_, *ω_B_*_2_, *ω_B_*_3_).

**Figure 3:**
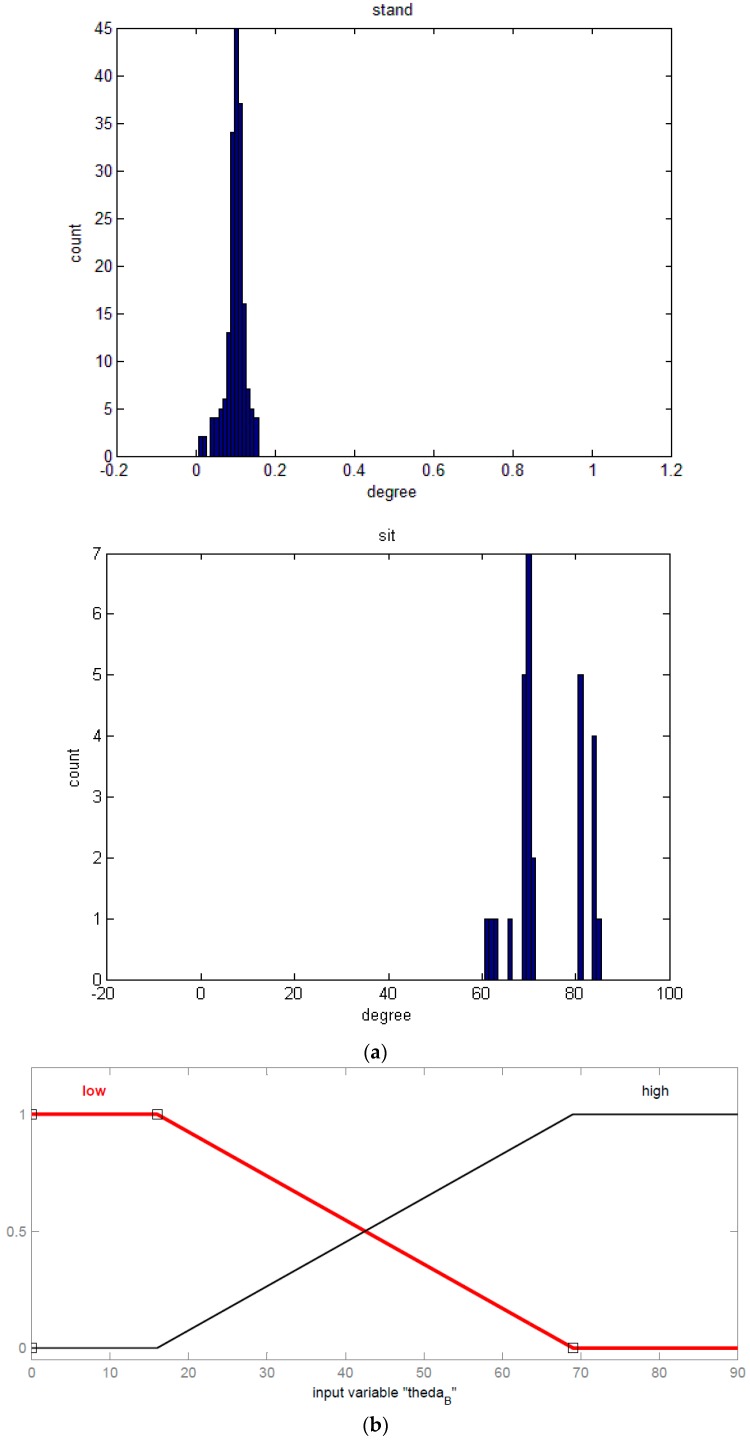

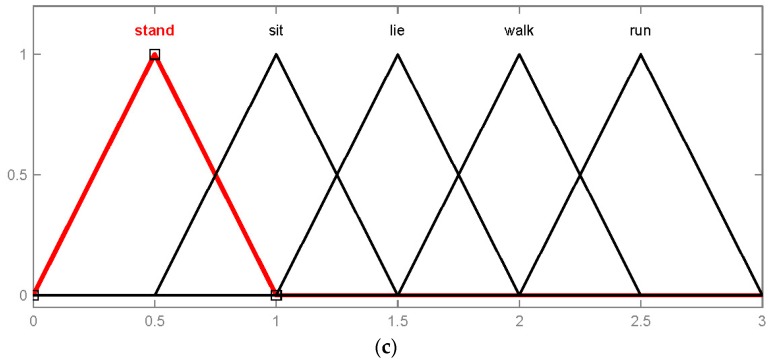
Distribution of measured signals of WSN sensor with respect to the fuzzy set of the feature in activity recognition: (**a**) distribution sample of probability density function of tilt angle measured by mote B (*θ_B_*) for “stand” and “sit” postures; (**b**) membership functions of input feature *θ_B_*; (**c**) membership functions of output features for defuzzification.

In practice, primary acceleration components are experienced in the *x*- and *z*-axes for forward-moving behaviors. Similarly, the angular velocity along the *x*- and *y*-axes is significant for the motions of body sway. Based on the specification of the sensor, the sensed data return random variables in form of probability density functions (PDF) which the output histogram follows Gaussian distributions as shown in Figure 3a. Therefore, we gathered a set of sensed data distributed during a period (e.g., 1 s), and took their mean value as the actual measured data.

### 2.2. Movement Features

The fuzzy computing process was utilized in the study to recognize the assigned movements. We initially suggested the native features of the motion, such as the measured accelerations and angular velocities, for the parameters to begin the fuzzy inference procedure.

1. *g_AR_* and *g_BR_*: the relative acceleration vectors for sensors A and B, respectively. The relative acceleration of motion can assist in detecting the starting motion when the body begins moving from the static position. Because of gravity, the initial acceleration of sensors in static status should be identical to 1 g. Thus, the relevant vectors for calculations were obtained by subtracting the initial acceleration from the present data, i.e., *g_AR_* = *g_A_* − *g_A_*_0_ and *g_BR_* = *g_B_* − *g_B_*_0_, where g_A0_ and g_B0_ are the initial acceleration vectors measured by sensors A and B, respectively.
2. *ω_AR_* and *ω_BR_*: the relative angular velocity vectors of sensors A and B, respectively. They can aid in recognizing body rotations. An initial value was subtracted from each angular velocity to calculate the rotating components in three directions. These were *ω_AR_* = *ω_A_* − *ω_A_*_0_ and *ω_BR_* = *ω_B_* − *ω_B_*_0_, where *ω_A_*_0_ and *ω_B_*_0_ are the vectors of initial angular velocity measured by sensors A and B, respectively. In addition, we extensively considered some features that can be derived from the features above to be the candidates for the process.
3. *θ_A_* and *θ_B_*: the tilt angles of sensors A (at the chest) and B (at the thigh) with respect to the initial status. The acceleration vectors at the *i*-th time step, for example, *g_Ai_* and *g_Bi_* for the sensors A and B, can be applied to conduct the tilt angle of the sensor with respect to the initial position. The tilt angles of sensor A can be computed by cos(*θ_A_*) = (*g_Ai_*∙*g_A_*_0_)/(|*g_Ai_*||*g_A_*_0_|) and vice versa for the sensor B. Let *θ_AB_* be a tilt angle between two sensors, *θ_AB_* = cos^−1^((*g_A_*∙*g_B_*)/(|*g_A_*||*g_B_*|)) is within domain [0, π]. In which, the operator “∙” denotes an inner product of two vectors, and |*g_A_*| = (*g_A_*_1_*g_A_*_1_ + *g_A_*_2_*g_A_*_2_ + *g_A_*_3_*g_A_*_3_)^1/2^. Theoretically, *θ_A_* should approximate 90° when the position of sensor A is changed from the postures of *sit* or *stand* to *lie*; similarly, *θ_B_* should approach 90° when the position of sensor B is changed from *stand* to *sit* or *lie*. A small angle is observed from *θ_B_* between sit and lie because sensor B on the thigh cannot be perfectly horizontal to the ground. Low, medium, and high degrees of the angle features can be defined for fuzzification.
4. *σ_gA_*, *σ_gB_*, and *σ_ω__A_*, *σ_ω__B_*: the standard deviation of acceleration and angular velocity of the chest and thigh, respectively. When the movement is unstable (e.g., *walk* or *run*), the sensed data may return significant variations in acceleration. Similarly, the shoulders and limbs sway when moving, creating angular velocity. Thus, these features aid in distinguishing the motion statuses of *walk* and *run*, if the body continues in a uniform motion without significant acceleration. The mean value (*μ*) and standard deviation (*σ*) of the PDF can be counted to evaluate moving levels.
  (1) $$\sigma ={\left\{\frac{1}{n}\sum_{i=1}^{n}{\left(x_{i}-\mu \right)}^{2}\right\}}^{\frac{1}{2}}$$
  where $\mu =\frac{1}{n}\sum_{i=1}^{n}x_{i}$ and x_i_ is each record of the measured data vector x that contains n records.
5. ν_gAR_ and ν_gBR_: the difference rate of relative acceleration of the chest and thigh, respectively, to the initial status. Similar to extracting the standard deviation, these features given by Equation (2) return normalized differences in the accelerations at the *i*-th time step with respect to the initial status. Particularly, apparent variations of the features extracted from the sensor B were studied for dynamic motion of the thigh.
  (2) $$v={\left|V_{i}-V_{0}\right|}^{2}/{\left|V_{0}\right|}^{2}$$
  where, vector V*_i_* is the parameter measured by the specified sensor at the i-th time step, and *V*_0_ is the initial value. They both have components in the three axes.
6. *γ_gA_*, *γ_gB_* and *γ_ω__A_*, *γ_ω__B_*: the gradient of acceleration and angular velocity of the chest and thigh, respectively. For either sensor A or B, each gradient component of acceleration or angular velocity on the 1-, 2-, and 3-axes at the *i*-th time step can be given by Equation (3).
  (3) $$\gamma_{gi}=\frac{g_{i}-g_{i-1}}{\Delta t},\;\gamma_{\omega i}=\frac{\omega_{i}-\omega_{i-1}}{\Delta t}$$

In which ∆t is the time interval between the (*i* − 1)-th and *i*-th time steps, and *g_i_* and *ω_i_* are the mean value of measured acceleration and angular velocity at the *i*-th time step, respectively. If the movement is severe, then the absolute value of gradient is large with respect to the others.

### 2.3. Fuzzy Inference System

**Figure 4:**
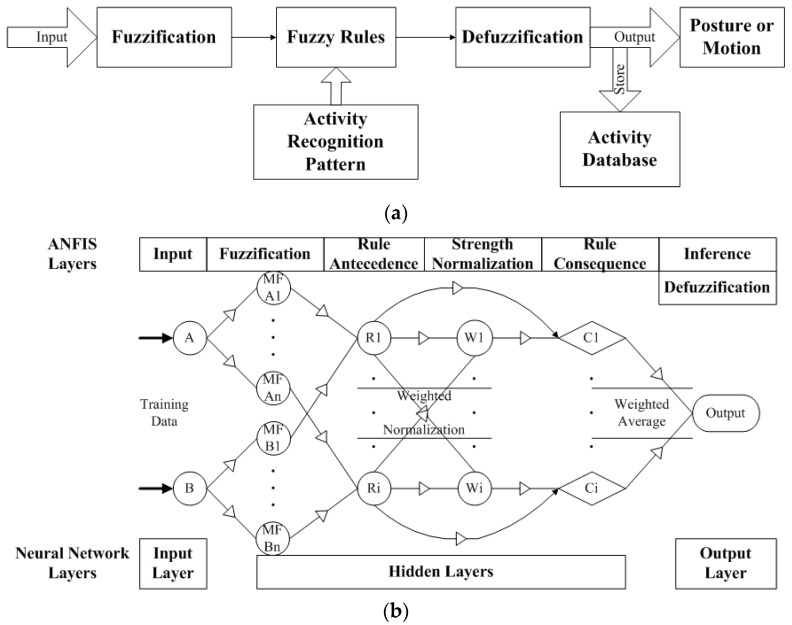
Fuzzy computing procedures for activity recognition: (**a**) the fuzzy inference process; (**b**) ANFIS modeling nodes based on fuzzy inference system and neural network layers.

These features are chosen for the inference rules of a fuzzy logic algorithm to reciprocally compute measured data and evaluate their feasibility. The process as shown in Figure 4a initially classifies proper features for the fuzzy sets that may include different types of the membership functions to fuzzificate the input features in the FIS. After fuzzification, if-then-based rules that control fuzzy logic between the input and output features are defined to yield activity patterns. Consequently, output features can be inferred from the movements according to the defuzzification process, and the fuzzy computing model can be generated for activity recognition. The inference procedure is described in the following steps.

(i) Select qualified input and output features required by the algorithm. Proper features are adopted by comparing the variations of the feature values and practical activities. The various inputs can be collaborated reciprocally for a similar output. For example, {*θ_A_*, *θ_B_*, *ω_BRx_*, *g_BRx_*, *σ_gBx_*} can be combined as a set of input features for SET-1 to get the output feature of activities, such as *lie*, *sit*, *stand*, *walk*, and *run*. Similarly, an input feature set, such as {*θ_A_*, *θ_B_*, *γ_ω__Ax_*, *γ_gBx_*, *σ_gAx_*} for SET-2, could yield equivalent outputs but based on different criteria, where subscript x denotes the x-axis component in the global coordinates.
(ii) Create corresponding membership functions due to the input features for fuzzification that define the participative degrees of the features in the activity. It allows various distribution criteria of membership functions in the FIS. For example, *θ_B_* can present significant low- or high-angle degrees in two PDFs of *stand* and *sit* as shown in Figure 3a. In this case, Figure 3b plots the membership functions that the trapezoidal distribution was simply applied for the FIS.
(iii) Induce the fuzzy rules for activity recognition. These rules are created by the fuzzy logic of “if-then” syntax to recognize the input features and conduct the output features; for example, if (*θ_B_* = “high-angle”, *θ_A_* = “low-angle” and *ω_BRx_* = “low” neglecting *g_BRx_* and *σ_BRx_*) then the output = *sit*. The membership function of output features for activities, such as *lie*, *sit*, *stand*, *walk*, and *run*, can be quantified by using the triangle distribution, as shown in Figure 3c, for the Mamdani-type FIS.
(iv) Substitute the fuzzy rules with the input and output features into the defuzzification process to produce resultant patterns of recognition. One set of fuzzy rules may yield a pattern criterion, whereas the corresponding output feature can be obtained by the given input features.

### 2.4. Adaptive Neuro-Fuzzy Inference System

The adaptive neuro-fuzzy inference system (ANFIS) was studied in the proposed model to enhance computing on the dynamic motions that performed the quite irregular variation of data with respect to static postures as shown in Figure 2. The ANFIS typically hybridizes benefits of FIS and neural network, and includes six layers that are input, fuzzification, rule antecedence, rule strength normalization, rule consequence, and inference with defuzzification [37]. In which, with the process as shown in Figure 4b, the fuzzification layer allows the clustering algorithm to allocate input variables for an initial fuzzy set, and the antecedent layer constructs the nodes that represent the membership functions. During the training cycles, the antecedent parameters (i.e., membership functions) will be modified with the fuzzy rules in the strength normalization layer iteratively until the root mean square errors (RMSEs) of the training sets are going steady. Then, the consequent layer combines them to determine the degree of the output. Thus, the rule-related layers above implicate the hidden layer with respect to the neural network. Finally, the node of inference layer computes the crisp output with defuzzification. In this study, we thus employed the ANFIS modeling of MATLAB™, which the output feature (i.e., constant weighted average) was involved in the Sugeno-type fuzzy system to enable the training process as detailed below.

(i) Define the motion index of the output feature. In this study, we defined a range of arbitrary values for the indexes of dynamic motions; e.g., let the random numbers belong to the index range [1, 2] and [3, 4] (e.g., 1.65 and 3.63), and represent *walk* and *run*, respectively.
(ii) Assign membership functions for the chosen features of input. For this case, we chose three components of *σ_gB_* as input features and assigned Gaussian-type membership function to the fuzzy set. In which, if there are m features for input and n membership functions for each feature, then the ANFIS modeling requires m^n^ constants for output features. Therefore, we simply used 3 Gaussian membership functions for each input feature and 27 weighted-average numbers for the crisp output to create the initial FIS.
(iii) Load training data set. A column of the motion indexes was added to the data set of the input feature, and we can load them with the initial FIS into the ANFIS for training. In addition, the applied toolbox supports the functionality of grid partitioning for automatically generating the fuzzy set of the initial FIS according to the loaded data.
(iv) Repeat the training process till steady. The ANFIS can adapt the necessary parameters of the initial FIS in the training process and return the RMSEs of each epoch. The process needs to be repeated until the RMSEs reach a steady value. Consequently, a final FIS can be obtained for estimating the output.

With the training procedure, the ANFIS produce appropriate fuzzy logic rules of the FIS that would be able to recognize the combinative activities.

## 3. Recognition Procedure and Results

We designed serialized actions that are processed by standard postures and motions for practicing the recognition procedure with FIS and ANFIS modeling, and assigned limited rules that can be extended as criteria in UHC measurement. Each subject followed the requests to sit for 10 s, stand for 10 s, run approximately 16 m in 6 s, walk about 16 m in 10 s, and then lie for 10 s (excluding the interval of several seconds between changing each action). The testers wore the WSN sensors on specified positions to observe the sample motions. The movements remained on the same plane and were conducted in a straight line. Variations of acceleration and angular velocity of activities are diagramed in the PDF histogram, and the parameters of these features can be calibrated. According to this procedure, we initially assigned seven and three testers respectively in the sample and blind-test groups to evaluate the FIS model. Then, we recruited eight testers, who are different from the previous ones but repeated the same procedure to retrieve dynamic motions of *walk* and *run* for yielding the training data set in the ANFIS model. Consequently, three other testers walked and ran with similar speed for testing. Regarding the FIS model, the sample groups modeled the FIS rules and reached 100% recognition rate. We thus applied the model in a blind test, which the testers were asked to repeat the procedure arbitrarily but were not restricted by the specific action order. The greatest difference between two tests is that irregular (or unorderly) movements would affect the rate of successful recognition in the blind test. We adopted two representatives (i.e., SET-1 and 2) from several tests to describe the proposed recognition method. Moreover, the data training process was learned in the ANFIS model for improvement.

### 3.1. Membership Functions and Fuzzification

The first step of fuzzification is finding the membership function. We considered *θ_A_* and *θ_B_* to recognize the static postures *lie*, *sit*, and *stand* and used *σ_gA_* or *σ_ωB_* for the dynamic motions of *walk* and *run*. In this test, the static posture was *sit* if *θ_A_* and *θ_B_* were approximate to 0° and 90°, respectively. If *θ_A_* did not change significantly but *θ_B_* increased to a low angle, then the posture could be *stand*. In contrast, if *θ_A_* changed significantly but *θ_B_* did not, then the posture was *lie*. Thus, a suitable range of angles can be adjusted for different postures. For example, if *θ_A_* is less than a low angle, such as 15°, it probably represents *sit* and *stand*; if *θ_B_* is about 15° to 30°, it might represent *lie* because the subject’s thigh is not parallel to the posture in sit.

Furthermore, the features *σ_gA_* or *σ_ω__B_* were observed to produce impulsive amplitudes with respect to nearby time steps during a movement period; they would help judge when the motion is changed from static to dynamic and vice versa. However, this movement status was excluded because it is not important to recognize the beginning of *walk* or *run* for the scope of this study. Therefore, *σ_gA_* determines movement status, such as static postures and dynamic motions. For example, the activity is observed to be static or dynamic when *σ_gA_* is approximately 0 g, greater than 0.1 g, or between 0 and 0.1 g. In addition, *σ_ω__B_* implies a swaying condition of the thigh for *walk* and *run*. For instance, a proper range of *σ_ω__B_* can be discerned to see if *walk* and *run* are less and greater than 25°/s, respectively. As considering other feature sets, *θ_A_* and *θ_B_* can be used to recognize static postures, but (*ν_gAR_*, *ν_gBR_*) or (*γ_ω__A_*, *γ_gB_*) can be used to aid in judging dynamic motions (e.g., *walk* and *run*). Using this concept from the previous feature set, the corresponding membership functions can also be defined for further analysis.

In the FIS, the successfully-received data of each static posture or dynamic motion would be inspected to plot the PDF histogram for determining the membership functions of the features as mentioned in previous section. For these static postures, the measured features were scattered as the Gaussian distribution typically with a significant peak (e.g., Figure 3a). We then simply applied a set of trapezoidal functions including low-, median-, or high-angle degrees, in which the angle with respect to the peak of distribution is the height of the trapezoid. Considering irregular distribution of the features for dynamic motions, the minimum and maximum values of the features are typically used as the criterion boundaries of the trapezoidal function in the study. The suggested boundary cuts of membership functions with respect to the adopted features are shown in Table 1, where the suffixes *x*, *y*, or *z* are the components on the corresponding axes. The membership functions can be formulated as Ф(*Λ*) = {[*f_n_*(*x*)|D*_n_*(δ_0*n*_,δ_1*n*_)]*_n_*} for these features; thus,

(4) $$\Phi (\Lambda )=\{{\left[f_{n}(x)|\delta_{0n}\leq x\leq \delta_{1n}\right]}_{n}\},n=1\text{ to }m$$

where Ф(*Λ*) is a membership function of feature *Λ*, which contains m stepwise functions *f_n_*(*x*) in domain D*_n_*. For the domain of the *n*-th distribution, the lower and upper limits are given by δ_0*n*_ and δ_1*n*_, respectively. The symbol δ implies boundary values of the distribution functions related to the features. The neighboring distribution functions in the FIS are permitted to overlap each other. For example, the upper limit δ_11_ of the first distribution of Ф(*θ_A_*) could be greater than the lower limit δ_02_ of the second one when they are overlapped, and can perhaps depict a changing action. These boundary parameters lead to different strengths of the corresponding membership functions (i.e., from 0 to 1), which yield the criteria of the fuzzy set. In this study, the stepwise trapezoidal functions were adopted to present the degrees of activity features corresponding to data distribution. For example, variations in the feature *θ_B_* contribute low-angle degrees for the *sit* posture with respect to high-angle degrees for *stand*; its membership function includes two trapezoidal functions for both angles. The low-angle function has several cuts: the boundaries *δ*_01_ = 0 and *δ*_11_ = 16 with strength of 1 for the cut from 0° to 16°, *δ*_02_ = 16 and *δ*_12_ = 36 with strength from 1 to 0 (or, [1→0]) for the cut from 16° to 36°, and *δ*_03_ = 36 and *δ*_13_ = 90 with 0 of strength for the cut above 36°. The high-degree function shows that (*δ*_01_, *δ*_11_) = (0, 30) with strength 0, (*δ*_02_, *δ*_12_) = (30, 60) with strength from 0 to 1, and (*δ*_03_, *δ*_13_) = (60, 90) with strength 1. Similarly, the membership functions of other features can be determined by their fuzzy cuts. SET-1 and 2, {*θ_A_*, *θ_B_*, *ω_BRx_*, *g_BRx_*, *σ_gBx_*} and {*θ_A_*, *θ_B_*, *γ_ω__Ax_*, *γ_gBx_*, *σ_gAx_*}, respectively, are proposed for the scope.

Successively, the membership functions of output features can be generated for the FIS when the input features have been created. We thus considered triangular distributions *g_n_*(*x*) given by Equation (5) to assign five membership functions of the output feature for the activities *lie*, *sit*, *stand*, *run*, or *walk*.

(5) $$\Phi (\Lambda )=\{{\left[g_{n}(x)|\delta_{0n}\leq x\leq \delta_{1n}\right]}_{n}\},n=1\text{ to }5$$

where, the boundary parameter *δ* can be defined as (*δ*_0*n*_, *δ*_1*n*_) = (*n* − 1, *n*) for *g_n_*(*x*).

The parameters of membership functions relative to the chosen features are shown in Table 1 to generate the fuzzy logic rules for later defuzzification.

### 3.2. Fuzzy Logic Rules and Defuzzification

Using the MATLAB™ toolbox, the fuzzy logic rules can be computed in the defuzzification process that employs the classical vertex method [50]. Fuzzy logic for this study thus involves several combination sets of rules, which are shown in Table 2. For example, the rule for the stand posture is as follows: if *θ_A_* is low, *θ_B_* is low, and *ω_BRx_* is low; then, the output is stand.

**Figure 5:**
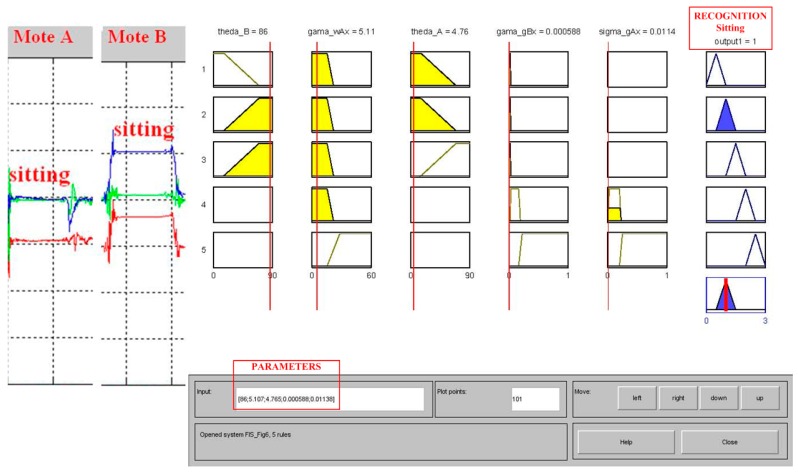
Substitution of fuzzy parameters for activity recognition.

By substituting the fuzzy set into the rules, the output can be inferred at the centroid of distributions according to the defuzzication process, and the cut value of the output movement can be obtained from the FIS model. For example, in Figure 3c, the centroids of {*stand*, *sit*, *lie*, *walk*, *run*} are {0.5, 1, 1.5, 2, 2.5}, respectively. With the FIS model, the arbitrary arguments of input features can be inputted to estimate participation in the output features based on the fuzzy rules, and to conduct the qualified movements. For example, as shown in Figure 5, if the arguments of the feature set {*θ_A_*, *θ_B_*, *γ_ωAx_*, *γ_gBx_*, *σ_gAx_*} from the measured data are substituted by {4.76, 86, 5.11, 0.000588, 0.0114}, respectively, then the output value is 1, which is in the range of (0.5, 1.5) of the motor membership function and is recognized as a *sit* action.

### 3.3. Results from FIS Modeling

According to the assigned activity, each tester in the sample group would deliver about 50 records from measurement excluding changing actions. After generating the FIS model, the parameters of each feature set, which represents the action at a time point, can be substituted into the model to recognize an inferential posture or motion. We thus applied the FIS in the blind test to infer the movement data of the testers (i.e., about 50 records for each one) and counted the successful rates as shown in Table 3. As results, recognition rates of the static postures versus the dynamic motions were (99%:84%) and (99%:93%) respectively for SET-1 and 2, overall.

In the blind test, the testers repeated the sample motions but not restricted to the same order and the identical behavior (i.e., the irregular movements with personal habits or changing poses would probably cause unsatisfactory recognition rates). Thus, the stable and regular movements can be successfully recognized according to the designed FIS. The results would present accuracy of the developed devices and feasibility of the proposed procedure in this study. However, the personal habits of the testers could be inconsistent with regularity of the dynamic motions by the sample group and affect the accuracy of recognition. That is, if the testers follow the assigned process in the rehabilitation program, their movements can be correctly caught and traced by the robotic facility. If not, we need to improve the recognition process. We rearranged the sample group for the sample test procedure, and found that the dynamic motions required remodeling the FIS for better recognition accuracy. The improvement result is discussed in next section. For the UHC applications, the behaviors exclusive (or unrecognized) from the assigned activity can provide useful information to monitor incorrect motions that are out of the threshold in rehabilitation.

### 3.4. Improvement with ANFIS Modeling

**Figure 6:**
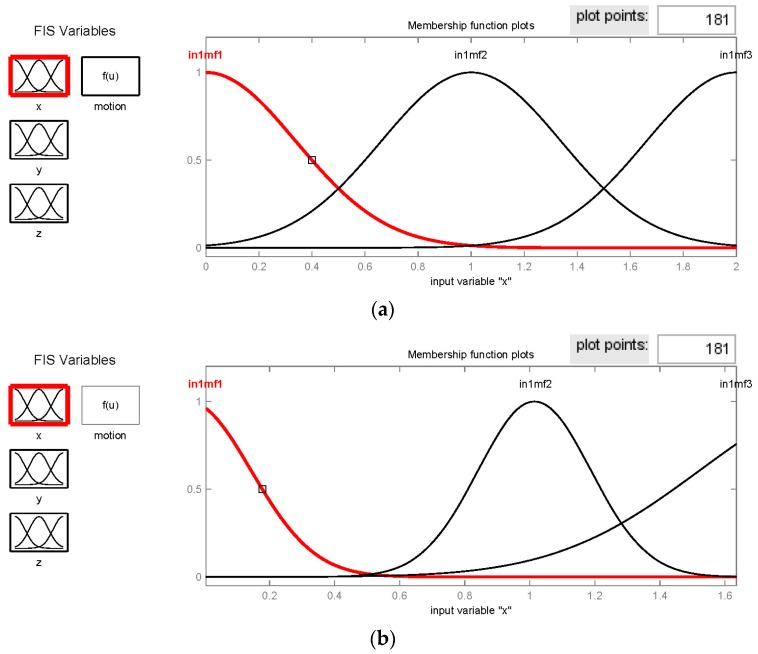

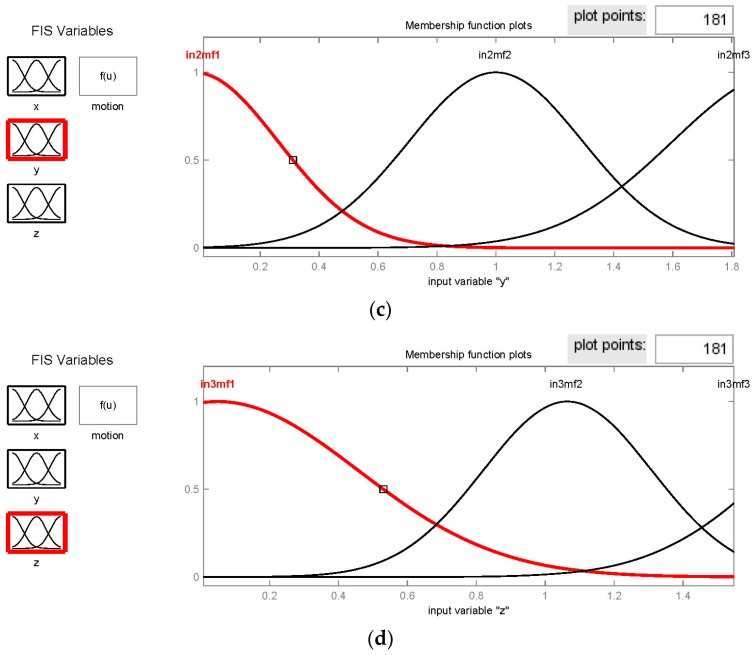
Membership functions (MFs) in the fuzzy sets of the initial and trained FISs for recognizing the motions of *walk* and *run*: (**a**) identical MFs of three features for the initial FIS; (**b**) adapted MFs of feature x; (**c**) adapted MFs of feature y; and (**d**) adapted MFs of feature z for the trained FIS; where, in1mf1 represents the first membership function of the first input feature (i.e., x), and is similar for three input features. The (*x*, *y*, *z*) means three components of the standard deviation of acceleration by the thigh (*σ_gB_*) for this example.

Furthermore, we employed the ANFIS modeling to improve recognition performance for the dynamic motions that were not accurately computed in the designed FIS model. In the ANFIS, a total of about 200 records were counted for *walk* and *run*, but 187 records without change-motions were extracted from eight sample testers for the training process. Following the procedure in the previous section, the initial and trained FIS, as shown in Figure 6, were verified by a blind test.

We initially selected three components of σ_gB_ as the input features in the range of [0, 2], and assigned three Gaussian-type membership functions with identical standard deviation 0.3397 but mean at 0, 1, and 2 for each feature, as shown in Figure 6a. In addition, we separated the output range in [1, 2] and [3, 4] by 26 constants that distribute 13 equal intervals of 0.0769 within each range, and allocated the 27th constant at 2.5 if not in both ranges (or, unrecognizable motion). According to this design, the crisp outputs in [1, 2] and [3, 4] respectively determine the motions of *walk* and *run*, and that in (2, 3) can indicate the uncertain motions. After a 40-epoch training process, we obtained the well-trained FIS as shown in Figure 6b–d that illustrated the adapted fuzzy set for inference; where, the relevant parameters are demonstrated in Table 4. However, the trained Sugeno-type FIS contains the out-of-range variables for some crisp outputs; e.g., mf6 = −242.3 or mf11 = 187.5 is not in the defined range from 1 to 4. It implicates the exclusive conditions if very different examples from training inputs are given for inference. Besides, the computing time of 40 epochs for training approximately 200 records of data sets (i.e., 200 points per set) was about 5–10 s.

**Figure 7:**
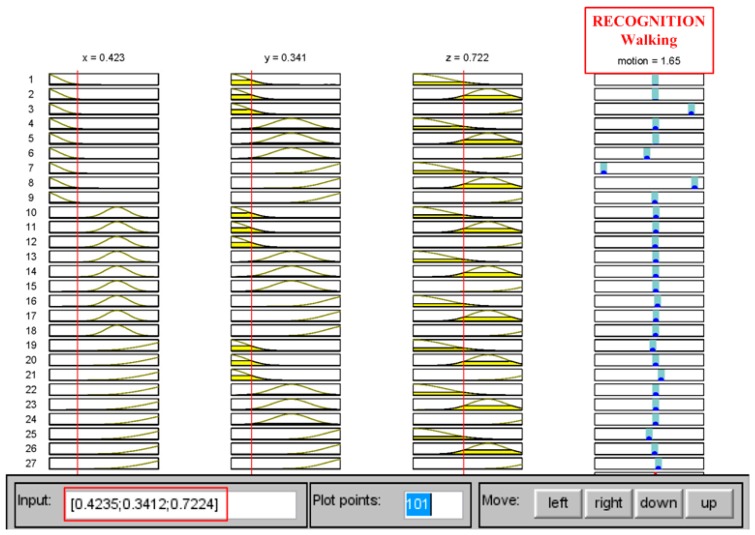

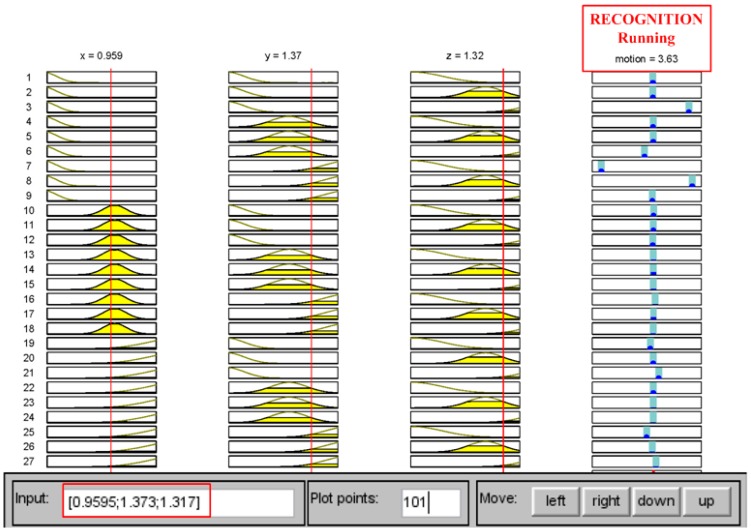
Recognition results from the trained FIS for the motions of walk and run, which are indexed within a range of [1, 2] and [3, 4], respectively.

Figure 7 shows the screen snapshot that inputs a set of feature values taken from the blind test. That is, the set (0.4235, 0.3412, 0.7224) and (0.9595, 1.3726, 1.3168) returned 1.61 and 3.31 that allocate in the range of *walk* and *run*, respectively. As predicting the movement, we imported the motion data of each blind-test user into the trained FIS to check the recognition rates. As a result, the blind test for three testers performed the effective recognition as shown in Table 4. In which, there was no mis-recognition case that occurred for both *walk* and *run* (i.e., the model did not recognize *walk* as *run*, and vice versa), and the rates would reach 100% if the uncertain output (because of ambiguous inputs) were ignored.

## 4. Discussion and Application

In this study, the laboratory members wore the self-developed WSN sensors on their chests and thighs to measure motor data for recognition. According to the assigned activity procedure, the sample-group members obeyed the serialized movements to deliver data for the FIS with the appropriate membership functions and the fuzzy logic rules. The FIS reached a 100% recognition rate for the sample group, and then was verified in the blind test that was processed by other members. The approaches with improvement are discussed below, and the proposed fuzzy computing model is expected for application in the UHC measurement.

### 4.1. Discussion on Fuzzy Computing Model

Many past studies for activity recognition used various algorithms to approach the anticipative outputs with the appropriate inputs. It is empirically known that the rule-based fuzzy logic is helpful to identify regular-steady models, e.g., pattern classification and decision tree analysis [51,52,53]; and the machine-learning method provides advanced algorithms, e.g., artificial neural networks and neural-fuzzy systems to trace irregular movements [54,55,56]. For instance, an earlier study considered the x component of acceleration to compare the accuracy of popular machine learning algorithms for measuring daily activities, which include sitting, standing, walking, running, climbing stairs, etc. The accuracies of collected data from the subjects would reach more than 90% if the training and testing data were in the same day; otherwise, the performance would be even less than 50% if they were measured in different days [49]. It implies that irregular movements would impact accuracy if both training and testing data were acquired from the different activity patterns. Regarding the fuzzy algorithm, the past study employed FIS to distinguish the activities for going downstairs, jumping, going upstairs, and moving forward with accuracy between 93% and 100% [40]. In addition, another study applied FIS, and compared it with some machine learning methods for tracing human activities of daily living—such as making a phone call, hand washing, cooking, and eating—by various sensor types, and reached the classification rate of about 94% [41]. According to the results of the proposed fuzzy computing models, we learned that the procedure of generating the FIS with proper input features was useful to recognize static postures, and the process improved by the ANFIS would be efficient for evaluating dynamic motions. Combinative feature sets can be adopted for generating the appropriate fuzzy system to compute a variety of movement data. The typical type of membership function, such as triangle or trapezoid function, was used in the Mamdani-type FIS, and the Gaussian function was employed in the ANFIS. As modeling the ANFIS, besides the input features, the recognizable activity can be denoted by index numbers in the training data set, in which the range-type index is suggested for the dynamic motion. The ranges of adaptive parameters in the Sugeno-type FIS are changed after the training process, and it probably leads out-of-range or uncertain outputs for unrecognizable testing data or ambiguous inputs such as changing actions. Effective recognition rates for the assigned movements can be approached in the blind test. Thus, the proposed FIS and ANFIS models with proper features can be further applied to assist activity recognition in physical therapy for UHC.

The prior study reviewed the designs of statistical pattern recognition, and concluded that the necessary factors for successful recognition include pattern classes, sensing environment, feature extraction, training and test samples, and performance evaluation [57]. In the practical rehabilitation design, the motor behavior typically includes asymmetry and complexity to cause measurement deviation. The earlier studies suggested the multi-sensor solution to detect activity by wearing twelve accelerometers on the body [58,59]. With a limited budget for hardware costs at the developing stage, the difficulty above would impact the measurement process. Thus, the pilot study considered combinative features to learn the potential features from the components of acceleration and angular velocity, and designed the movements with restriction to filter deviations in different axes. In other words, if the motor behavior can be the combination of several simple divided actions—e.g., separating the continuous *lie*-*sit*-*stand*-*walk*-*run* behavior into five unique actions—then one or two sensors were feasible to measure each action in the full activity process. Recently, a wearable sensor such as a sport bracelet is viable to decrease the hardware cost, and the solutions of multi-sensor and combinative features can both be expected in the fuzzy computing model to recognize more complicated activities at the next stage.

### 4.2. Application in Ubiquitous Healthcare

**Figure 8:**
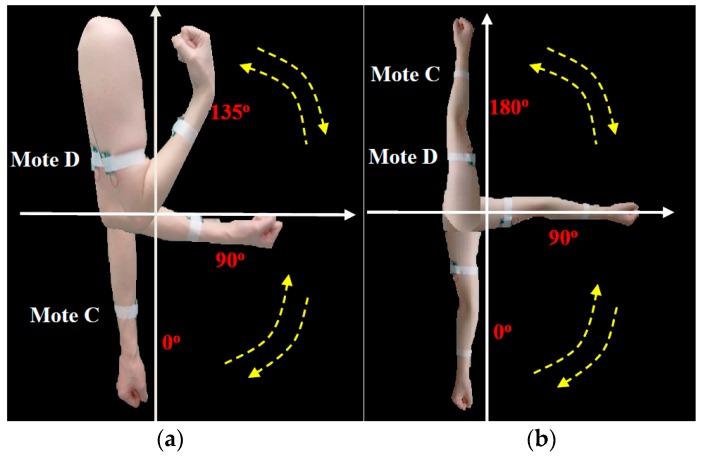
Flexion test for physical therapy: (**a**) flex the elbow (left); (**b**) raise the arm (right).

**Figure 9:**
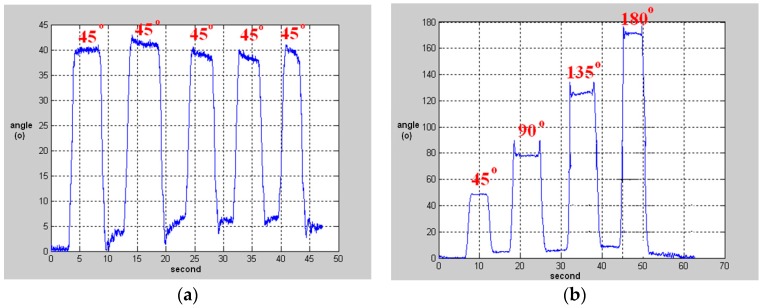
Variations of tilt angles between two motes for (**a**) flexing to the elbow (left); (**b**) raising the arm (right) [60].

The proposed model can be further extended to rehabilitation management for physical therapy in UHC. We applied a simple flexion exercise that was practiced in our previous study [60] with the recognition process for the UHC measurement to approve its feasibility. The test includes two upper-limb actions: (a) flex the elbow (0° → 135°), and (b) raise the arm in four steps (0° → 45° → 90° → 135° → 180°), as shown in Figure 8. The tester wore the sensors C and D at the wrist and the upper arm respectively, and processed each action steps every five seconds for counting the exercising times. The variations of the tilt angle features are plotted on Figure 9.

The feature set {*θ_C_*, *θ_D_*}, similar to the fuzzy logic of above {*θ_A_*, *θ_B_*}, represented the tilt angles between two motes for various action types. The angles recognized in each step were assessed by the thresholds {40°–45°, 85°–90°, 130°–135°, 175°–180°}. Table 5 presents the eligible actions with recognized angles against necessary counts of the actions. For instance, five and four steps were requested by actions (a) and (b), respectively, but only four and two motions were counted in successful passes. That is, if the movement was not recognized at the correct position, then the tester properly did not obey the therapy in a self-management program.

**Figure 10:**
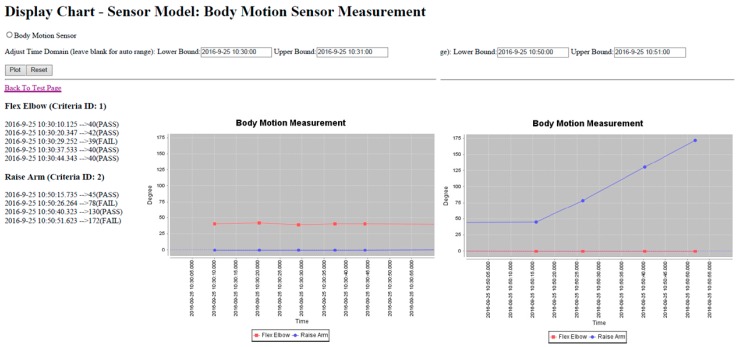
Web-based interface for UHC measurement to monitor flexion exercises “flex the elbow” and “raise the arm” in different periods.

We thus created a web-based interface by applying the criteria of physical therapy to monitor both movements. According to the prototype, we deployed WSN motes to transport the sensed data from the user who wore the self-developed WSN sensors. The gateway then sent the filtered data to the sink log of the backend server through the Internet. The model installed in the server activated the recognition process, and the records regarding the assigned movement were written to a data log. The packet was in form of “n|time|motion_1|motion_2” that denotes serial number of motion, recording time, degree of raising wrist, and lifting arm, respectively. For example, the packet “1|2016-9-25 10:30:00|40|−1” stands for the motion #1 that counted 40° for “flex elbow” but no “raise arm” (i.e., with negative value “−1”). Figure 10 presents a screenshot of the monitoring interface according to the test data in Table 5. The activity history of the assigned movements during the selected period can be reviewed by online diagrams, and the recognized motions by the FIS can be labeled by “pass” or “fail” based on the criteria. If the motion is not recognizable in the FIS, then the “fail” status will be marked because the motion features exceed the threshold.

Finally, the proposed fuzzy computing model can be approved by the simple flexion test in physical therapy, and is feasible for measurement applications in ubiquitous healthcare. For instance, the proposed model can be progressed with manual design of a personalized FIS model and process automation of activity recognition in the practical rehabilitation exercise of UHC. In which, the user can follow a personalized exercise process in the clinic that is designed by the physical therapist to provide a sample data set for training in the ANFIS model. Then, the trained FIS can be created within the web-based UHC system for real-time checking of the exercise data that are sent from a remote site at home. The mobile apps can be further joined with the system to transport the data through web services.

## 5. Conclusions

This study proposed a convenient process to measure body activities for UHC in physical therapy by using self-developed wearable WSN sensors. When the user wears the WSN sensor in movement, six components of acceleration and angular velocity can be concurrently acquired, and the movements can be recognized by the fuzzy computing model. The Mamdani-type FIS is employed in the model to calculate the sensed data for the recognition process. The input features optionally combine tilt angles, standard deviations, difference rates, and gradients for acceleration and angular velocity to determine the membership functions. Fuzzy logic rules are then defined to control the output features that index the movements including static postures and dynamic motions. Then, use of fuzzification and defuzzification can process recognition to output the indexes of movements. Two sample feature sets were designed in the FIS model for the assigned movements, and good recognition rates for static postures were reached in the blind test. Furthermore, an ANFIS model was studied to improve recognition for the dynamic motions. A Sugeno-type FIS was initially created by using features such as three components of acceleration at thigh. After the training process, the trained FIS approached the effective recognition rate, and the model would perform a feasible process for recognizing human activity. Finally, the proposed model was approved by two upper-limb exercises in physical therapy. A prototype of web-based interface was thus created to practice activity measurement for UHC. In practice, the recognition rate can represent the passing threshold of the assigned exercises. This approach contributed a fuzzy computing model with a noninvasive and wearable facility to help measure necessary rehabilitative movements of patients in physical therapy for UHC. In the future, the wearable sensor would be replaced by new devices with a wider measurement range of the modern accelerometer and gyroscope for advanced study at the next stage.

## Figures and Tables

**Table 1:** Suggested parameter range of the trapezoidal membership functions of input features for the proposed two fuzzy sets.

**SET-1**			
Feature Sets	[D_1_]:(*δ*_10_, *δ*_11_)	[D_2_]:(*δ*_20_, *δ*_21_)	[D_3_]:(*δ*_30_, *δ*_31_)
Ф(*θ_A_*)_low_ & Ф(*θ_B_*)_low_	[1]:(0, 16)	[1→0]:(16, 36)	[0]:(36, 90)
Ф(*θ_A_*)_high_ & Ф(*θ_B_*)_high_	[0]:(0, 30)	[0→1]:(30, 60)	[1]:(60, 90)
Ф(*ω_BRx_*)_low_	[1]:(0, 25)	-	-
Ф(*ω_BRx_*)_high_	[1]:(60, 300)	-	-
Ф(*g_BRx_*)_low_	[0→1]:(0, 0.08)	[1]:(0.08, 0.28)	[1→0]:(0.28, 0.4)
Ф(*g_BRx_*)_high_	[0]:(0, 0.28)	[0→1]:(0.28, 0.38)	[1]:(0.38, 1.35)
Ф(*σ_gBx_*)_low_	[0→1]:(0, 0.07)	[1]:(0.07, 0.28)	[1→0]:(0.28, 0.32)
Ф(*σ_gBx_*)_high_	[0→1]:(0.28, 0.4)	[1]:(0.4, 1)	-
**SET-2**			
Feature Sets	[D_1_]:(*δ*_10_, *δ*_11_)	[D_2_]:(*δ*_20_, *δ*_21_)	[D_3_]:(*δ*_30_, *δ*_31_)
Ф(*θ_A_*)_low_ & Ф(*θ_B_*)_low_	[1]:(0, 16)	[1→0]:(16, 36)	[0]:(36, 90)
Ф(*θ_A_*)_high_ & Ф(*θ_B_*)_high_	[0]:(0, 30)	[0→1]:(30, 60)	[1]:(60, 90)
Ф(*γ_ωARx_*)_low_	[1]:(0, 15)	[1→0]:(15, 22)	[0]:(22, 60)
Ф(*γ_ωARx_*)_high_	[0]:(0, 15)	[0→1]:(15, 30)	[1]:(30, 60)
Ф(*γ_gBRx_*)_low_	[1]:(0, 0.02)	[1→0]:(0.02, 0.0.3)	[0]:(0.03, 0.1)
Ф(*γ_gBRx_*)_medium_	[0→1] :(0, 0.02)	[1]:(0.02, 0.18)	[1→0]:(0.18, 0.2)
Ф(*γ_gBRx_*)_high_	[0]:(0, 0.18)	[0→1]:(0.18, 0.23)	[1]:(0.23, 1)
Ф(*σ_gAx_*)_low_	[0→1]:(0, 0.02)	[1]:(0.02, 0.2)	[1→0]:(0.2, 0.23)
Ф(*σ_gAx_*)_high_	[0→1]:(0.2, 0.3)	[1]:(0.3, 1)	-

**Table 2:** Fuzzy logic rules with respect to both feature sets for defuzzification.

Rule 1						Rule 2					
*θ_A_*	*θ_B_*	*ω_BRx_*	*g_BRx_*	*σ_gBx_*	Output	*θ_A_*	*θ_B_*	*γ_ωAx_*	*γ_gBx_*	*σ_gAx_*	Output
L	L	L	-	-	*stand*	L	L	L	L	-	*stand*
L	H	L	-	-	*sit*	L	H	L	L	-	*sit*
H	H	L	-	-	*lie*	H	H	L	L	-	*lie*
-	-	H	L	L	*walk*	-	-	H	M	L	*walk*
-	-	H	H	H	*run*	-	-	H	H	H	*run*

**Table 3:** Successful rate (%) of activity recognition by designed FIS.

Feature Set	*Lie*	*Sit*	*Stand*	*Walk*	*Run*	Average
Sample Test						
SET-1	100	100	100	100	100	100
SET-2	100	100	100	100	100	100
Blind Test						
SET-1	96	100	100	86.68	81.55	92.85
		(static posture:dynamic motion) = (99:84)				
SET-2	98	100	100	96.31	90.48	96.96
		(static posture:dynamic motion) = (99:93)				

**Table 4:** Antecedent parameters corresponding to the ANFIS modeling and recognition rate of the assigned dynamic motions estimated by the trained FIS.

Initial Status								
Input range: [0, 2]								
(*σ*, *μ*) of three Gaussian MFs ^(1)^ for each input feature: (0.3397, 0), (0.3397, 1), and (0.3397, 2)								
Output range: [1, 4]								
mf1	mf2	mf3	mf4	mf5	mf6	mf7	mf8	mf9
1.0769	1.1538	1.2308	1.3077	1.3846	1.4615	1.5385	1.6154	1.6923
mf10	mf11	mf12	mf13	mf14	mf15	mf16	mf17	mf18
1.7692	1.8462	1.9231	2.0000	3.0769	3.1538	3.2308	3.3077	3.3846
mf19	mf20	mf21	mf22	mf23	mf24	mf25	mf26	mf27
3.4615	3.5385	3.6154	3.6923	3.7692	3.8462	3.9231	4.0000	2.5000
After training process								
Input range			mf1 (σ, μ)		mf2 (σ, μ)		mf3 (σ, μ)	
In1: [0.0045, 1.6348]			(0.1941, −0.0506)		(0.1736, 1.0145)		(0.4448, 1.9665)	
In2: [0.0076, 1.8080]			(0.2840, −0.0230)		(0.2951, 0.9999)		(0.3830, 1.9823)	
In3: [0.0077, 1.5483]			(0.4087, 0.0492)		(0.2451, 1.0645)		(0.3420, 1.9982)	
Output range: [1.01, 4]								
mf1	mf2	mf3	mf4	mf5	mf6	mf7	mf8	mf9
1.6017	1.0093	171.5	2.1715	2.8963	−242.3	4.5227	3.4755	3.5141
mf10	mf11	mf12	mf13	mf14	mf15	mf16	mf17	mf18
−38.4	187.5	11.7	3.1452	3.6483	−0.9198	−1.855	0.9098	3.6164
mf19	mf20	mf21	mf22	mf23	mf24	mf25	mf26	mf27
3.3686	4.1074	2.7958	−10.763	28.7	3.7789	2.1909	15.8998	−28.038
Recognition rate (%) of dynamic motions by (*σ_gBx_*, *σ_gBy_*, *σ_gBz_*).								
**Group**	**Walk**		**Run**	**Record**		**Epoch**	**Uncertain ^(2)^**	**RMSE ^(3)^**
Training	-		-	187		40		0.4182
Testing 1	91.7 (100) ^(4)^		100 (100)	26			1	0.3889
	(11/12) ^(5)^		(14/14)					
Testing 2	100 (100)		92.3 (100)	24			1	0.3322
	(11/11)		(12/13)					
Testing 3	90 (100)		88.9 (100)	28			3	0.3386
	(9/10)		(16/18)					

Notes: ^(1)^ The Gaussian distribution function: $f(x;\sigma ,\mu )=e^{\frac{-{\left(x-\mu \right)}^{2}}{2\sigma^{2}}}$; ^(2)^ The uncertain data mean the output in the unrecognizable range in (2, 3) or out or the range; ^(3)^ The testing RMSEs excluding uncertain data; ^(4)^ Recognition rate for including (excluding) uncertain data; and ^(5)^ Correct counts/total counts.

**Table 5:** Recognition results of the simple flexion exercises in physical therapy [60].

Feature	Action Type	Recognized Angle (Degree)	Threshold (Degree)	Pass/Count
*θ_C_*	(a)	40, 42, 39, 40, 40	40~45	4/5
*θ_D_*	(b)	45, 78, 130, 172	{40~45, 85~90, 130~135, 175~180}	2/4

Note: the recognized angle values with underline stand for the correct activities.
